# Robotic transcranial magnetic stimulation motor maps and hand function in adolescents

**DOI:** 10.14814/phy2.14801

**Published:** 2021-04-04

**Authors:** Adrianna Giuffre, Ephrem Zewdie, Helen L. Carlson, James G. Wrightson, Hsing‐Ching Kuo, Lauran Cole, Adam Kirton

**Affiliations:** ^1^ Calgary Pediatric Stroke Program Alberta Children’s Hospital Calgary Alberta Canada; ^2^ Department of Pediatrics Cumming School of Medicine University of Calgary Calgary Alberta Canada; ^3^ Department of Clinical Neurosciences Cumming School of Medicine University of Calgary Calgary Alberta Canada; ^4^ Department of Physical Medicine & Rehabilitation University of California Davis CA USA

**Keywords:** motor development, motor mapping, neurophysiology, transcranial magnetic stimulation

## Abstract

**Introduction:**

Transcranial magnetic stimulation (TMS) motor mapping can characterize the neurophysiology of the motor system. Limitations including human error and the challenges of pediatric populations may be overcome by emerging robotic systems. We aimed to show that neuronavigated robotic motor mapping in adolescents could efficiently produce discrete maps of individual upper extremity muscles, the characteristics of which would correlate with motor behavior.

**Methods:**

Typically developing adolescents (TDA) underwent neuronavigated robotic TMS mapping of bilateral motor cortex. Representative maps of first dorsal interosseous (FDI), abductor pollicis brevis (APB), and abductor digiti minimi (ADM) muscles in each hand were created. Map features including area (primary), volume, and center of gravity were analyzed across different excitability regions (R100%, R75%, R50%, R25%). Correlations between map metrics and validated tests of hand motor function (Purdue Pegboard Test as primary) were explored.

**Results:**

Twenty‐four right‐handed participants (range 12–18 years, median 15.5 years, 52% female) completed bilateral mapping and motor assessments with no serious adverse events or dropouts. Gender and age were associated with hand function and motor map characteristics. Full motor maps (R100%) for FDI did not correlate with motor function in either hand. Smaller excitability subset regions demonstrated reduced variance and dose‐dependent correlations between primary map variables and motor function in the dominant hemisphere.

**Conclusions:**

Hand function in TDA correlates with smaller subset excitability regions of robotic TMS motor map outcomes. Refined motor maps may have less variance and greater potential to quantify interventional neuroplasticity. Robotic TMS mapping is safe and feasible in adolescents.

## Graphical abstract

We aimed to show that neuronavigated robotic TMS motor mapping in adolescents could efficiently produce discrete maps of individual upper extremity muscles, the characteristics of which would correlate with motor behavior. Healthy adolescents completed bilateral mapping and motor assessments with no serious adverse events or dropouts. Hand function in TDA correlates with specific regions of robotic TMS motor map outcomes and may have less variance and greater potential to quantify interventional neuroplasticity.

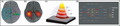

## 1 INTRODUCTION

Cortical stimulation can explore human motor function. In 1937, Penfield used electrical stimulation to map the organization of the motor cortex, defining the human homunculus (Penfield & Boldrey, 1937). These individualized topographical maps of the sensorimotor system are thought to be “plastic” with their organization influenced by development, genetics, experience, and injury (Sanes et al., 1988, 1990; Teskey et al., 2002). Motor maps may also be modifiable through occupational or physical therapy, pharmacology, or noninvasive neurostimulation, creating therapeutic relevance in persons with neurological disability (Barker et al., 1985; Pascual‐Leone, Nguyet, et al., 1995).

Animal models have informed our understanding of motor map physiology (Kaas et al., 1979; Snow et al., 1988). Somatotopic arrangement includes both precise control of individual muscles and overlapping representations of muscles, allowing for intermuscular and interjoint coordination for synergistic actions (Dechent & Frahm, 2003; Plow et al., 2010). In adult primates, precise tactile stimulation of one or two digits demonstrated how the sensory cortex can be selectively altered (Jenkins et al., 1990), and revealed correlations between cortical area representations and recent experiences (Recanzone et al., 1992). Similar evidence of functional plasticity occurs in the motor cortex (Rioult‐Pedotti & Donoghue, 2003). Peripheral nerve lesions, repetitive electrical stimulation, and behavioral motor training can rapidly alter cortical motor representations (Nudo et al., 1996; Sanes et al. 1992). Similar explorations of motor cortex somatotopy and its reorganization in humans may improve understanding of individualized motor function and plasticity.

Transcranial magnetic stimulation (TMS) can safely produce in vivo maps of the motor cortex (Barker et al., 1985; Lefaucheur & Picht, 2016; Rossi et al., 2009; Wassermann et al., 1992). Typical TMS motor map outcomes include area or volume, measure of cortical excitability, and center of gravity (COG) (Rioult‐Pedotti & Donoghue, 2003). These mapping metrics can be used to quantify motor cortex plasticity such as enlarged map areas or shifts in COG (Wassermann et al., 1992; Wilson et al., 1993). TMS motor maps may also be able to quantify changes in cortical motor representations associated with motor learning (Cohen et al., 1993; Pascual‐Leone, Nguyet, et al., 1995). Translational applications of TMS motor mapping include preoperative neurosurgical planning for brain tumor removal and epilepsy surgery (Lefaucheur & Picht, 2016; Picht et al., 2011).

Despite the utility of TMS motor mapping, applications in children have been limited. Pediatric populations may provide ideal models to study motor map physiology given the plasticity of the developing brain in health and disease. TMS is safe and well tolerated in children with our program alone delivering over 4 millions stimulations to over 400 children without any serious adverse events (Zewdie et al., 2020). In children, TMS can quantify motor cortex neurophysiology including motor maps, in both healthy populations and those with neurological disabilities such as cerebral palsy (Garvey & Gilbert, 2004; Grab et al., 2018; Zewdie et al., 2017). For example, a study of children with hemiparetic cerebral palsy secondary to unilateral early brain injuries defined distinct motor maps in each hemisphere that appeared to change in response to therapy (Friel et al., 2017). Additional investigations of cortical maps in children with cerebral palsy found significant differences in cortical representation sites for hand and forearm muscles between healthy children and children with CP, though COG and optimal stimulation sites were not reported (Maegaki et al., 1999). A lack of motor mapping studies in the typically developing brain currently limits the utility of this approach in pediatric populations.

Multiple challenges in both motor mapping and pediatric TMS may be overcome by emerging technologies. Motor thresholds are much higher in young children, making it difficult to obtain consistent motor‐evoked potentials (MEP) (Garvey & Gilbert, 2004). TMS motor mapping is time‐consuming with multiple potential sources of error and variance. Typical protocols using manual TMS are long and may be imprecise due to human errors. Robotic TMS, synchronized with MRI‐based neuronavigation systems may overcome many of these challenges. Robotic TMS reduces acquisition time and provides consistent coil positioning in three dimensions (Ginhoux et al., 2013). Near real‐time motion correction also accommodates subject movement. This may be an advantage in children where we have shown robotic TMS is both feasible and well tolerated (Giuffre et al., 2019; Grab et al., 2018; Julkunen, 2014; van de Ruit & Grey, 2016).

Finally, navigated robotic TMS may facilitate the collection of detailed motor maps including efficient analysis of multiple muscles at various excitability levels (Massé‐Alarie et al., 2017; Uy et al., 2002). The effect of varying stimulation intensities and muscle activation on COG (mm) has been explored but has not been investigated using subset excitability regions of motor maps (van de Ruit & Grey, 2016).M1 excitability regions and maps have been associated with specific motor tasks (Massé‐Alarie et al., 2017; Uy et al., 2002) but this approach remains unexplored in children and adolescents. The borders of motor maps may also be most variable, introducing a potential source of noise (Brasil‐Neto et al., 1992). To address this issue, more restricted excitability “subsets” of motor maps have been considered (Uy et al., 2002). Such selected regions of excitability within M1 motor maps may also relate to motor function (Massé‐Alarie et al., 2017). However, subset excitability maps have not been investigated in the pediatric population.

We used robotic TMS to generate detailed bilateral motor maps of hand muscles in typically developing adolescents and compared their characteristics across hemispheres, muscles, and their relationship to motor performance.

## 2 METHODS

### 2.1 Participants and ethical approval

Typically developing adolescents were recruited via the Healthy Infants and Children Clinical Research Program (HICCUP), a population‐based research cohort. Inclusion criteria were: 1) age 12–18 years, 2) typical neurodevelopment, 3) right handed (as reported by the Edinburgh Handedness inventory with a laterality index greater than −28 and self/parent report) (Oldfield, 1971), 4) informed consent/assent, and 5) no contraindications to MRI and NIBS (Keel et al., 2001). Participants actively taking neuropsychiatric medications, or with developmental or neuropsychiatric diagnoses were excluded.

All participants consented to participate in the Accelerated Motor Learning in Pediatrics (AMPED) study which was a randomized, single‐center, double‐blind controlled interventional trial testing whether transcranial direct current stimulation (tDCS) and high‐definition (HD‐tDCS) could enhance motor learning, the results of which are reported elsewhere (Cole et al., 2018). This study describes and reports the robotic TMS motor mapping that was performed on Day 1 of the AMPED trial (Cole et al., 2018). Participants and their guardians provided informed consent and assent. The study was approved by the University of Calgary Research Ethics Board.

### 2.2 Magnetic Resonance Imaging

Images were obtained at the ACH Diagnostic Imaging Centre using a 3 T General Electric MR750w scanner (GE Healthcare) with a 32‐channel head coil using a fast‐spoiled gradient echo sequence (FSPGR BRAVO, 226 axial slices, TR = 8.5 ms, TE = 3.2 ms, voxels = 1 mm isotropic). Each participant's T1‐weighted image was entered into the TMS neuronavigation system (Brainsight2, Rogue Research) for subsequent motor mapping.

### 2.3 Robotic TMS Motor Mapping

**FIGURE 1:**
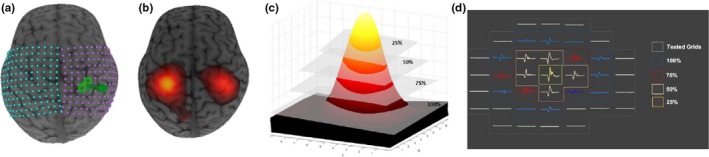
Visualization of robotic TMS M1 motor maps. (a) Grid (12 × 12, 7 mm) overlaid over T1‐weighted anatomical images, centered over right‐ and left‐hand knobs. (b) Overlay of computed right and left robotic TMS motor map area. (c) Subset excitability regions of motor map volume (R100%‐R25%). *X* represents the *x*‐axis on the 12 × 12 grid, whereas *Y* represents the *y*‐axis. (d) Representation of MEP amplitudes decreasing in size from R25% excitability region of the map to the perimeter of the map, excitability region 100%

Motor mapping was performed using a TMS robot (Axilum Robotics) within the ACH Pediatric Noninvasive Brain Stimulation Laboratory (Zewdie et al., 2020). Descriptions of the robotic motor mapping methods can be found elsewhere (Giuffre et al., 2019). Using an optical detection camera system (Polaris, NDI Medical Solutions), participants’ anatomical images were co‐registered with sensors. A figure‐of‐eight 70 mm Air‐Film coil (Magstim) accurately maintained its alignment and position in near real‐time (1 cm/second) (Ginhoux et al., 2013; Goetz et al., 2019; Grab et al., 2018). Using the neuronavigation software, a 12 × 12 rectangular grid with 7 mm spacing was superimposed on the reconstructed curvilinear brain and centered over the anatomical hand‐knob (Figure 1a) (Yousry et al., 1997). Each grid‐point trajectory was aligned tangentially to the cortical surface with coil position maintained at 45° in relation to the interhemispheric fissure.

Participants were seated in a comfortable chair with an option to watch a movie. Ag‐AgCl electrodes (Kendall) were placed on both hands over three muscles: first dorsal interosseous (FDI), abductor pollicis brevis (APB), and abductor digiti minimi (ADM) (Giuffre et al., 2019; Grab et al., 2018). A ground electrode was placed over the styloid process. MEP were captured using surface electromyography (EMG), amplified (×1000) using a CED 1401 signal analog/digital converter (Cambridge Electronic Design), filtered (20–2000 Hz), and digitized at 5000 Hz (Signal 6.0 software, Cambridge Electronic Design Limited) (Zewdie & Kirton, 2016).

Each robotic TMS session began with mapping of the right hemisphere. All motor mapping outcomes were based on the threshold of the contralateral primary target muscle (FDI) contralateral to the simulating hemisphere. The FDI “hotspot” was determined as the grid‐point that produced the largest, most consistent MEP. The resting motor threshold (RMT) of the left FDI (LFDI) was extrapolated from 5% of the slope of a stimulus–response curve (SRC) (Ridding & Rothwell, 1997; Temesi et al., 2014), this point was then used to determine the TMS mapping intensity. The motor mapping was done at the TMS intensity of 120%RMT (Ridding & Rothwell, 1997, 2007). Whenever the mapping intensity was greater than 100% maximum stimulus output (MSO), they were mapped at 100% MSO.

For each hemisphere, motor mapping began at the FDI hotspot grid‐point. Four single pulses over 4 seconds (1 Hz) were delivered at each grid‐point. A grid‐point was deemed “responsive” if ≥2/4 MEPs had peak‐to‐peak amplitudes at least ≥50 µV in any of the three‐hand muscles. From the grid‐point corresponding to the “hotspot,” the robotic system moved to each successive grid‐point, repeating the stimulation protocol. A snake‐like pattern continued along grid‐points until a “null point” was reached, generating the first border of the map. The map of one hemisphere was completed once null points formed a complete perimeter. To ensure a null point was a true “non‐responsive” point, border points were sampled twice.

### 2.4 Map analysis

Motor maps were analyzed using a custom mapping script (MATLAB R2016b, The MathWorks, Inc.). Motor maps were characterized by the following outcomes:

1. Area: Number of responsive points multiplied by the grid point area (7 mm × 7 mm = 49 mm^2^).
2. Volume: Averaged peak‐to‐peak MEP amplitude at each responsive point multiplied by the summated grid area.
3. Center of Gravity (COG): Weighted distribution of the largest MEP amplitude (Wassermann et al., 1992), in a 2D *x–y* plane assuming the *z* is equal to zero at the surface of the head. *Xi* and *yi* are the respective *x*‐, *y*‐ coordinates of the location where the peak‐to‐peak MEP amplitude (*Mi*) was recorded. $xCOG=\frac{\sum xiMi}{\sum Mi}\;yCOG=\frac{\sum yiMi}{\sum Mi}$
4. Euclidian distance of COGs in the *x*–*y* plane (2D): A straight‐line distance between two COGs between subset excitability regions (described below) within the same muscle (mm) assuming the two COGs have the same *Z* value.

### 2.5 Subset excitability regions

The additional step of measuring subset excitability regions of motor maps was investigated as the border of motor maps have typically been known to introduce greater variability (Brasil‐Neto et al., 1992) and that more restricted excitability regions of motor maps may reduce variability (Uy et al., 2002). Other studies have shown that different regions of excitability may relate to specific motor tasks (Massé‐Alarie et al., 2017). Specific excitability levels (Figure 1c) corresponded to MEP amplitudes collected at responsive grid points above a specific threshold (Figure 1d). Map borders were based on mean peak MEP amplitude (at the hotspot) multiplied by incremental percentiles of 25%, 50%, and 75%. The total excitability region of the map (R100%) included responsive grid points of MEP amplitudes ≥50 µV (represented by the blue MEP in Figure 1d) allowing map area (2‐dimensional) and volume (3‐dimensional) to be calculated. Subsequent excitability regions were calculated based on each participants’ individual maximum MEP amplitude (Figure 1c). For example, R75% represents all responsive grid points with MEP amplitudes >25% of the maximum MEP amplitude (see red MEP in Figure 1d). Accordingly, R25% represents the “peak of the motor map mountain” (Figure 1c).

### 2.6 Motor function assessments

Participants performed the Purdue Pegboard Task (PPT, primary), Jebsen Taylor Task (JTT), and Serial Reaction Time Task (SRTT) after robotic TMS mapping sessions. The PPT is a common motor assessment used to measure motor learning (Gardner & Broman, 1979; Tiffin & Asher, 1948). Participants placed as many pegs as possible into a pegboard in 30 seconds with the right‐hand (PPT_R_) and the left hand (PPT_L_). Each subtest was performed three times and scores were averaged. The JTT consists of seven subtests of common activities of daily living to measure unimanual hand function and was performed using their right‐hand (JJT_R_) and left‐hand (JTT_L_). Subtests include card flipping, picking up and dropping small objects, checker stacking, and grasping and releasing heavy and light cans (Jebsen et al., 1969). The first subtest (handwriting) was excluded. The final score was the summation of time to complete all subtests. The SRTT was performed using the left hand only, measuring reaction time in seconds (Honda et al., 1998; Nissen & Bullemer, 1987).

### 2.7 Safety and tolerability

Immediately following TMS mapping, participants completed a pediatric noninvasive brain stimulation safety and tolerability questionnaire (Zewdie et al., 2020). The robotic TMS mapping experience was ranked against common childhood experiences; 1) play a game, 2) birthday party, 3) watch TV, 4) long car ride, 5) go to dentist, 6) shot at the doctor, and 7) throwing‐up. Participants were also screened for symptoms of headache, neck pain, unpleasant tingling, light‐headedness, nausea, and any other self‐reported symptoms all of which were graded as mild, moderate, or severe.

### 2.8 Statistical analysis

Analyses were performed using the R statistical software package (RStudio Team, 2015). Motor map characteristics and motor scores were tested for normality using the Shapiro–Wilk test. Data are reported as mean and standard deviation (SD) unless otherwise stated. The coefficient of variation (CV = SD/mean) was calculated to examine the variability of different map characteristics. The CV is a ratio between the SD and the mean that affords a unique measurement of distribution variability that is insensitive to fluctuations in raw mean such that variability can be directly compared across distributions with varying means.

To determine the intra‐individual variability of the FDI motor maps between the largest (R100%) and smallest (R25%) subset excitability region, a CV was calculated from each participants’ MEP at responsive points (map area). Paired *t*‐tests were used to test for a difference in intra‐individual variability (CV) between the RFDI muscle at 100% and 25% (Wilcoxon‐signed rank test) and between the LFDI muscle at R100% and R25% (Paired *t*‐test).

Motor map outcomes across all subset regions were compared descriptively to confirm the expected patterns of change across percentiles. Once confirmed, subsequent analyses compared only the largest (R100%) and smallest (R25%) excitability thresholds to mitigate the effects of multiple comparisons. The false discovery rate was also corrected to control for multiple comparisons (Benjamini & Hochberg, 1995).

Repeated measures analysis of variance (RM ANOVA) tested for differences in motor map area and volume between muscles of the right and left hands using post hoc comparisons (Holm–Sidak corrected) following significant effects of muscle or hand dominance on motor map area and volume. To examine the effects of age and gender on associations between motor performance and motor map characteristics, correlations between the two were followed by robust linear regressions, with motor map characteristics, age, and gender as predictors (Wilcox, 2019). RM ANOVA tested for difference in Euclidian distances between subset excitability regions of FDI motor maps using post‐hoc comparisons (Holm–Sidak and Tukey corrected).

Spearman's ρ rank correlation coefficients were used to describe associations between motor performance and motor map characteristics. Paired samples *t*‐tests (Wilcoxon‐signed rank test) were used to compare motor map area, volume, and motor scores between the right and left hemisphere and hands.

## 3 RESULTS

### 3.1 Population Characteristics

Twenty‐four participants were recruited, and all completed the study (range 12–18 years, median 15.5 years, 52% female, Table 1). One participant was excluded as their motor mapping intensity exceeded 100% MSO resulting in incomplete MEP recordings. A further three participants received mapping of only the right hemisphere due to time constraints. This resulted in a final sample of 20 participants with unilateral maps (right hemisphere) and 23 participants with bilateral motor maps. The motor learning results from the AMPED trial are described elsewhere (Cole et al., 2018).

**TABLE 1:** Demographics

Participant	Right‐hand				Left‐hand					
	Age	Sex	PPT_R_	JTT_R_	Participant	Age	Sex	PPT_L_	JTT_L_	SRTT
01	17.26	M	15.33	17.84	01	17.26	M	15.00	19.89	0.63
02	17.08	M	14.33	21.87	02	17.08	M	13.00	23.97	0.57
03	16.04	F	16.67	17.36	03	16.04	F	13.33	18.62	0.42
05	15.83	M	15.67	17.04	04	17.17	F	15.33	18.4	0.47
06	13.78	F	16.33	18.17	05	15.83	M	13.33	19.95	0.46
07	14.89	M	11.00	20.06	06	13.78	F	15.67	20.89	0.56
08	14.41	M	15.00	21.46	07	14.89	M	12.00	20.27	0.62
09	18.92	F	19.00	17.22	08	14.41	M	13.00	26.76	0.58
10	16.44	F	14.33	17.36	09	18.92	F	16.00	19.03	0.36
11	16.95	F	16.00	20.39	10	16.44	F	14.67	21.25	0.51
12	16.25	F	15.67	23.41	11	16.95	F	13.00	22.64	0.48
13	15.26	F	14.67	19.06	12	16.25	F	11.67	25.4	0.54
14	14.25	F	13.67	25.45	13	15.26	F	13.67	21.19	0.59
15	14.68	M	15.67	19.89	14	14.25	F	13.33	28.78	0.50
16	14.76	M	12.67	22.22	15	14.68	M	13.00	20.75	0.51
17	17.51	F	15.33	20.49	16	14.76	M	12.67	24.32	0.71
18	17.06	F	17.33	21.87	17	17.51	F	15.33	22.61	0.45
20	12.40	M	17.00	19.05	18	17.06	F	16.33	21.58	0.50
21	16.28	F	17.33	17.11	19	13.37	M	11.67	22.11	0.62
23	14.79	M	16.67	24.21	20	12.40	M	14.67	18.4	0.51
					21	16.28	F	15.67	20.46	0.60
					22	14.44	M	11.67	25.96	0.50
					23	14.79	M	13.67	21.78	0.46
Mean	15.74	9 M:11F	15.48	20.08	Mean	15.64	11 M:12F	15.64	21.96	0.53
SD	1.49		1.74	2.53	SD	1.56		1.56	2.78	0.08

Demographics of the right‐hand analysis and left‐hand analysis. Motor scores on the PPT. Purdue Pegboard Task (PPT) in the right (PPT_R_) and left (PPT_L_) hand. JTT, Jebsen Taylor Task; JTT_R_ scores using the right‐hand. JTT_L_ scores using the left‐hand. SRTT, Serial Reaction Time Task. SRTT_L_ scores using the left‐hand. SD, Standard deviation

### 3.2 Mapping and Thresholds

A typical example of a motor map overlaid on a 3D anatomical brain with illustrations of the subset excitability regions is shown in Figure 1. A single unilateral motor map was produced in an average time of 17 (5.6) min, (total duration of 11–25 min), while bi‐hemispheric maps took an average of 34 (10.9) minutes (total duration of 21–52 min). Participants were encouraged to take breaks when needed and were given a break between hemispheres during bi‐hemispheric mapping.

RMT were comparable between right and left hemispheres, resulting in the mean RMT of 60.00 ± 0.1%MSO in the right hemisphere and 58.91 (0.1)%MSO in the left hemisphere. Mean hotspot magnitude (MEP amplitude) of the right hemisphere (1.95 (−1.9)mV) and left hemisphere (2.07 (1.8)mV) were also similar (*p* = 0.729).

### 3.3 Age and sex effects

Both sex and age were associated with multiple components of motor map thresholds and characteristics as well as motor function tests. Accordingly, all subsequent results reflect analyses using linear regression to correct for these effects. Age was not significantly correlated with RMT in the right hemisphere (*r* = −0.112, *p* = 0.612) nor in the left hemisphere (*r* = 0.116, *p* = 0.617).

### 3.4 Motor map area

**FIGURE 2:**
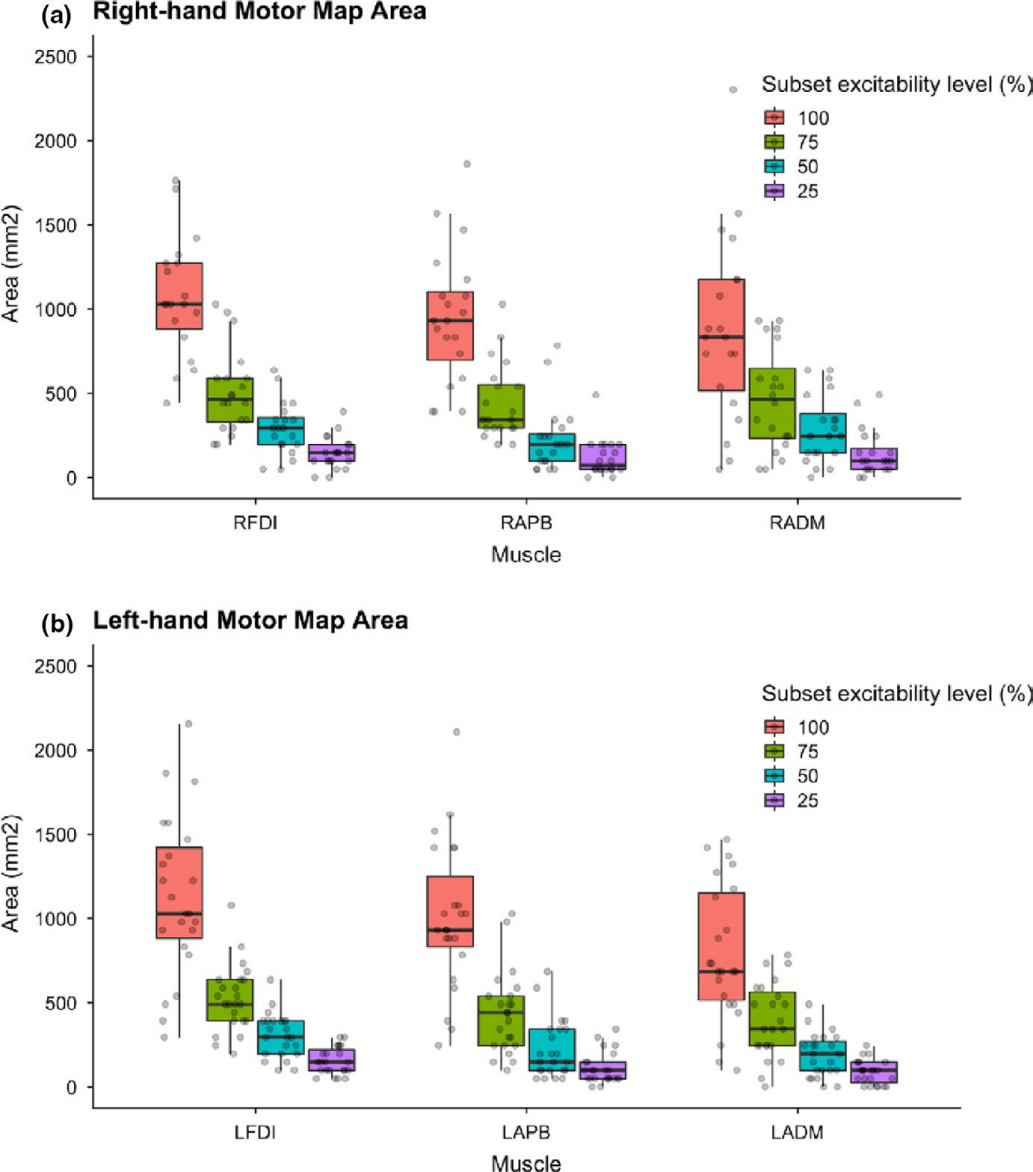
Subset excitability regions of motor map area in hand muscles. Motor map area (mm^2^) of the right‐hand (a) and left‐hand (b) muscles across subset excitability regions. Intra‐individual variability decreased as subset regions decreased from 100%‐25% of map area. FDI, First dorsal interosseous; APB, abductor pollicis brevis; ADM, abductor digiti minimi; Arbitrary subset excitability regions chosen (100% = pink), 75% = green, 50% = blue, 25% = purple)

Motor map area for all three muscles in both hands differed by subset excitability regions (Figure 2, Table 2) as expected such that the mean area was largest for the 100% and lowest for the 25% regions. Descriptive statistics for bilateral map areas are summarized in Table 2. For the tested muscle (right FDI), intra‐individual variability of MEP (map area) decreased from the largest (R100%) to smallest region (R25%) excitability region. Intra‐individual variability of map area decreased from the largest (R100%, CV = 1.261) to smallest (R25%, CV = 1.031) subset excitability regions (*Z* = −2.251, *p* = 0.024). Similarly, in the left FDI, intra‐variability of map area decreased in the left FDI (LFDI) from R100% (CV = 1.114) to R25% (CV = 0.977) (*t* = 2.623, *p* = 0.015).

**TABLE 2:** Descriptive statistics of right‐ and left‐hand motor map area

	R100%			R25%		
	FDI	APB	ADM	FDI	APB	ADM
Right‐hand Map Area (mm^2^)						
Mean (mm^2^)	1141.70	948.15	874.65	144.55	117.60	144.55
SD	477.22	396.89	551.92	97.32	111.74	135.34
CV	0.42	0.42	0.63	0.67	0.95	0.94
Left‐hand Map Area (mm^2^)						
Mean (mm^2^)	1127.00	1007.70	794.65	153.39	110.78	87.35
SD	477.82	436.73	406.48	79.98	93.69	72.31
CV	0.42	0.43	0.51	0.52	0.85	0.83
Right‐hand Map Volume (mm^2^/mV)						
Mean (mm^2^/mV)	810.90	389.80	224.91	294.65	112.80	68.55
SD	634.00	343.24	212.01	370.36	145.40	84.82
Left‐hand Map Volume (mm^2^/mV)						
Mean (mm^2^/mV)	831.75	407.18	164.11	272.14	108.30	33.09
SD	745.29	349.98	150.33	335.13	129.38	40.91

	Right‐hand Euclidean distance COG (mm)			Left‐hand Euclidean distance COG (mm)		
	R100‐75%	R100‐50%	R100‐25%	R100‐75%	R100‐50%	R100‐25%
Mean (mm)	0.26	0.47	1.34	0.25	0.40	0.73
SD	0.16	0.49	0.40	0.18	0.22	0.49

Descriptive statistics of motor map area (mm^2^) and motor map volume (mm^2^/mV) in the right‐hand and the left‐hand at two subset excitability regions (R100% and R25%). Euclidean distance of COG between subset excitability regions of the right and left FDI. Subset excitability region 100% and 25%. FDI, First dorsal interosseous; APB, abductor pollicus brevis; ADM, abductor digiti minimi; COG, Center of gravity; SD, Standard deviation; CV, Coefficient of variation.

Map areas of the right and left FDI muscle were comparable at the R100% (*Z* = −0.786, *p* = 0.432) and R25% (*Z* = −0.598, *p* = 0.550) excitability regions. Given map area of hand muscles was based on the FDI threshold, we expected that the representation of the FDI map area would be the largest compared to other muscles (Table 2). We did however find differences in the mean map area of right‐hand muscles between muscles and excitability regions. At the largest excitability region (R100%), the mean map area differed between right‐hand muscles (left hemisphere) (*F* = 10.800, *p* < 0.001). Positive differences were found between the mean map area of RFDI‐RAPB (*t* = 3.26, *p* = 0.005), and RFDI‐RADM (*t* = 4.50, *p* = 0.001). For the R25% excitability region, the mean area of right‐hand muscles did not differ (*F* = 0.846, *p* = 0.437) (Table 2). In the left‐hand muscles, the mean map area also differed across excitability regions (*F* = 11.000, *p* < 0.001). Positive Holm–Sidak analysis showed that positive differences were observed between LFDI‐LADM (*t* = 4.620, *p* < 0.001) but not LFDI‐LAPB (*t* = 1.66, *p* = 0.104) at the R100%. Mean area at the R25% excitability region differed between left‐hand muscles (*F* = 0.607, *p* = 0.005), specifically LFDI‐LADM (*t* = 3.44, *p* = 0.004) (Figure 2).

### 3.5 Motor map area and motor performance

**FIGURE 3:**
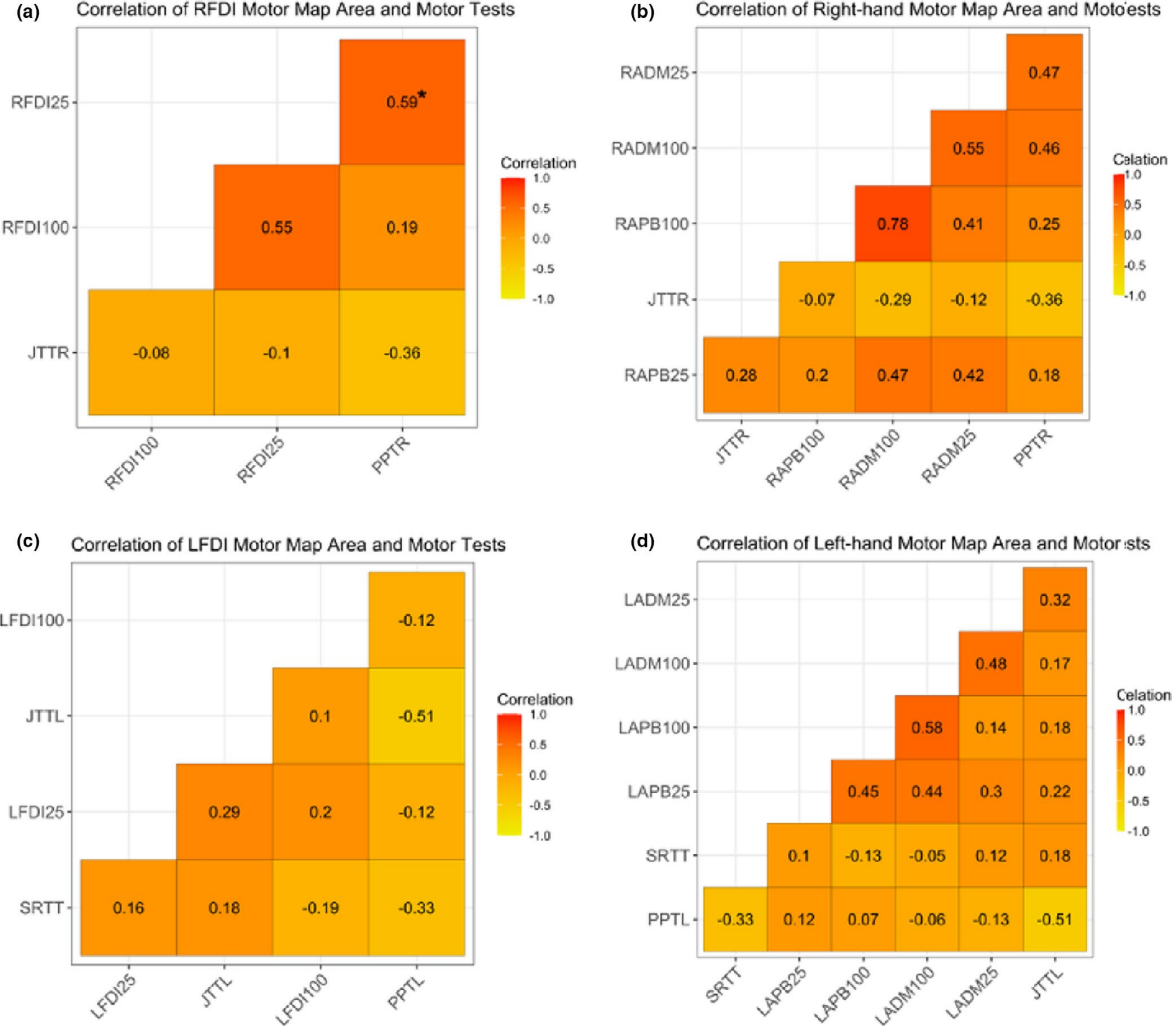
Correlations between motor map area at subset percentiles with motor tasks in the right‐ and left‐hand muscles. Correlations of subset percentiles of the right‐hand tested muscle (RFDI) map area and right‐hand motor scores (PPT_R_ and JTT_R_) (a) and of secondary right‐hand muscles map area and right‐hand motor scores (PPT_R_ and JTT_R_) (b). Correlations of subset percentiles of the left‐hand tested muscle (LFDI) map area and left‐hand motor scores (PPT_L_, JTT_L_, and SRTT) (c) and of secondary left‐hand muscles map area and left‐hand motor scores (PPT_L_, JTT_L_, and SRTT) (d). PPT_R_, Purdue Pegboard Task scores in the right‐hand; PPT_L_, Purdue Pegboard Task scores in the left‐hand; JTT_R_, Jebsen Taylor Task scores in the right‐hand; JTT_L_, Jebsen Taylor Task scores in the left‐hand; SRTT, Serial Reaction Time Task; FDI, First dorsal interosseous (tested muscle), secondary muscles; APB, abductor pollicis brevis; ADM, abductor digiti minimi. Arbitrary subset excitability regions chosen (R100%, R75%, R50%, R25%). *Significant correlations (*p* < 0.05). Heat map: Correlations 1.0 (red) to −1.0 (yellow)

Correlations between motor map area and PPT are summarized in Figure 3. Right FDI motor map area (left hemisphere) at the R100% did not correlate with right‐hand motor function (PPT_R_) (ρ = 0.192, *p* = 0.455, Figure 3a). However, RFDI map area at R25% was correlated with PPT_R_ scores including corrections for age and gender (ρ = 0.589, *p* = 0.032). This association was consistently observed in a dose‐dependent fashion across the interval regions of R75% and R50%. Map areas of the secondary right‐hand muscles did not correlate with PPT_R_ scores at any excitability region though rho values were consistently positive (Figure 3b).

In the right hemisphere, PPT_L_ scores did not correlate with left FDI map areas at either R100% (ρ = −0.115, *p* = 0.753) or R25% (ρ = −0.120, *p* = 0.753) excitability regions (Figure 3c). No correlations were observed between map area of secondary left‐hand muscles with PPT_L_ scores across subset excitability regions (Figure 3d).

### 3.6 Motor map volume

**FIGURE 4:**
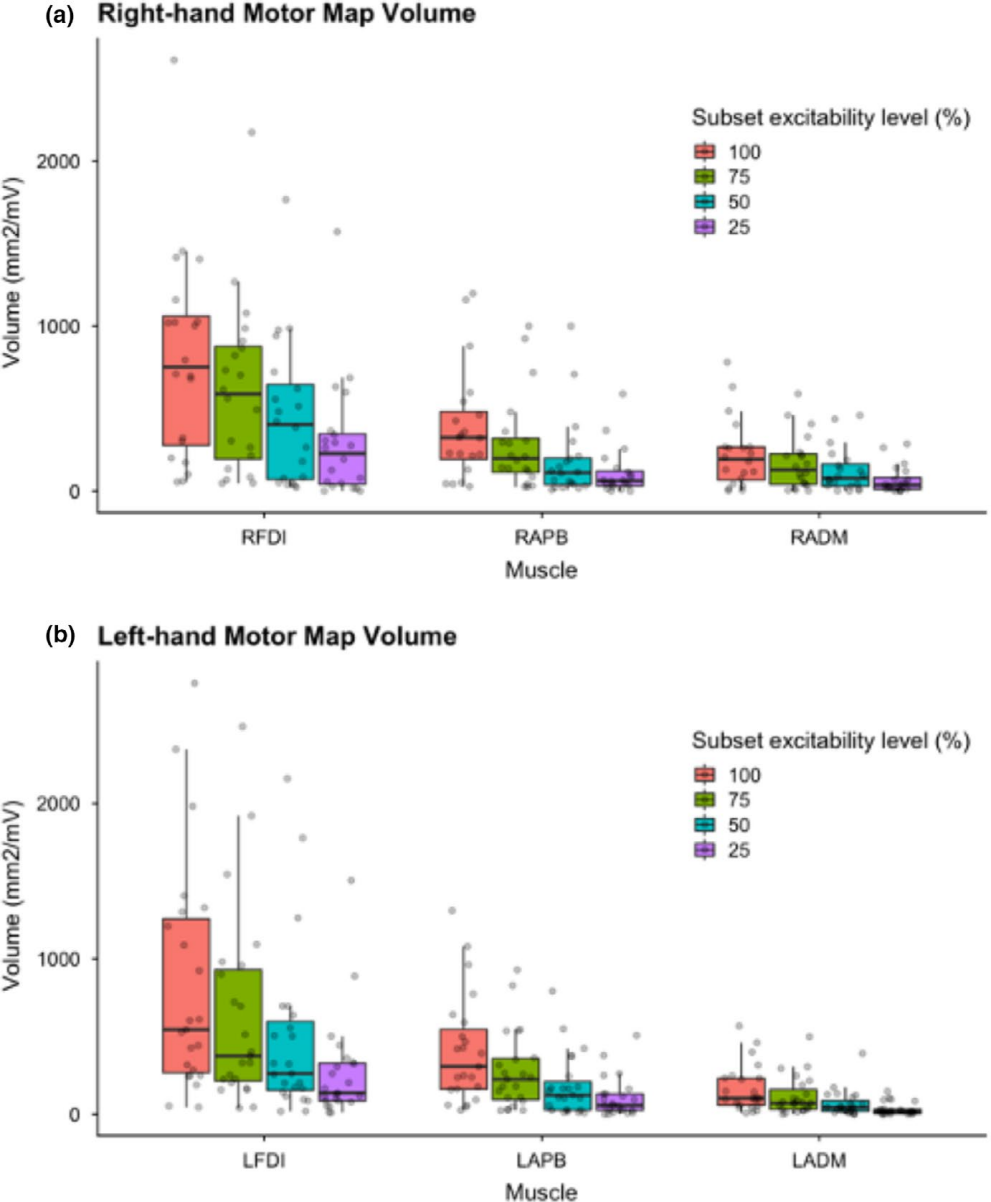
Subset excitability regions of motor map volume in hand muscles. Motor map volume (mm^2^/mV) of the right‐hand (a) and left‐hand (b) muscles across subset percentiles. FDI, First dorsal interosseous; APB, abductor pollicis brevis; ADM, abductor digiti minimi; Arbitrary subset excitability regions chosen (100% = pink), 75% = green, 50% = blue, 25% = purple)

Motor map volume for all three muscles in both hands also differed by subset excitability regions (Figure 4) such that mean volume was lowest for the R25% region maps. Descriptive statistics of map volume of the right‐ and left‐hands are reported in Table 2. In a similar fashion observed above for motor map area, smaller excitability regions of motor map volume were associated with decreased variance. Distributions of map volume variance in right‐ and left‐hand muscles are shown in Figure 4.

Map volumes of the right and left FDI were comparable between the two hemispheres at all excitability regions including R100% (*Z* = −0.709, *p* = 0.478) and R25% (*Z* = −0.597, *p* = 0.550). In the right‐hand muscles, mean map volume between muscles differed at the largest excitability region (R100%) (*F* = 23.8, *p* < 0.001) between RFDI‐RAPB (*t* = 6.680, *p* < 0.001), and RFDI‐RADM (*t* = 4.800, *p* < 0.001) but not RADM‐RAPB (*t* = −1.880, *p* = 0.068). For the R25% excitability region, mean volume of right‐hand muscles differed (*F* = 8.630 *p* = 0.004) between RFDI‐RAPB (*t* = 3.920, *p* < 0.001), RFDI‐RADM (*t* = 3.152, *p* = 0.006) but not between RADM‐RAPB (*t* = −0.767, *p* = 0.448). Mean map volume differed between all left‐hand muscles at the largest subset excitability region (R100%, *F* = 16.500, *p* < 0.001), LFDI‐LADM (*t* = 5.670, *p* < 0.001), LFDI‐LAPB (*t* = 3.610, *p* = 0.002), and LADM‐LAPB (*t* = −2.06, *p* = 0.045). Differences in mean map volume of left‐hand muscles were found at R25%,(*F* = 10.400, *p* = 0.002), between LFDI‐LADM (*t *= 4.46, *p* < 0.001), LFDI‐LAPB (*t* = 3.06, *p* = 0.008), but not between LADM‐LAPB (*t* = −1.40, *p* = 0.168).

### 3.7 Motor map volume and motor performance

**FIGURE 5:**
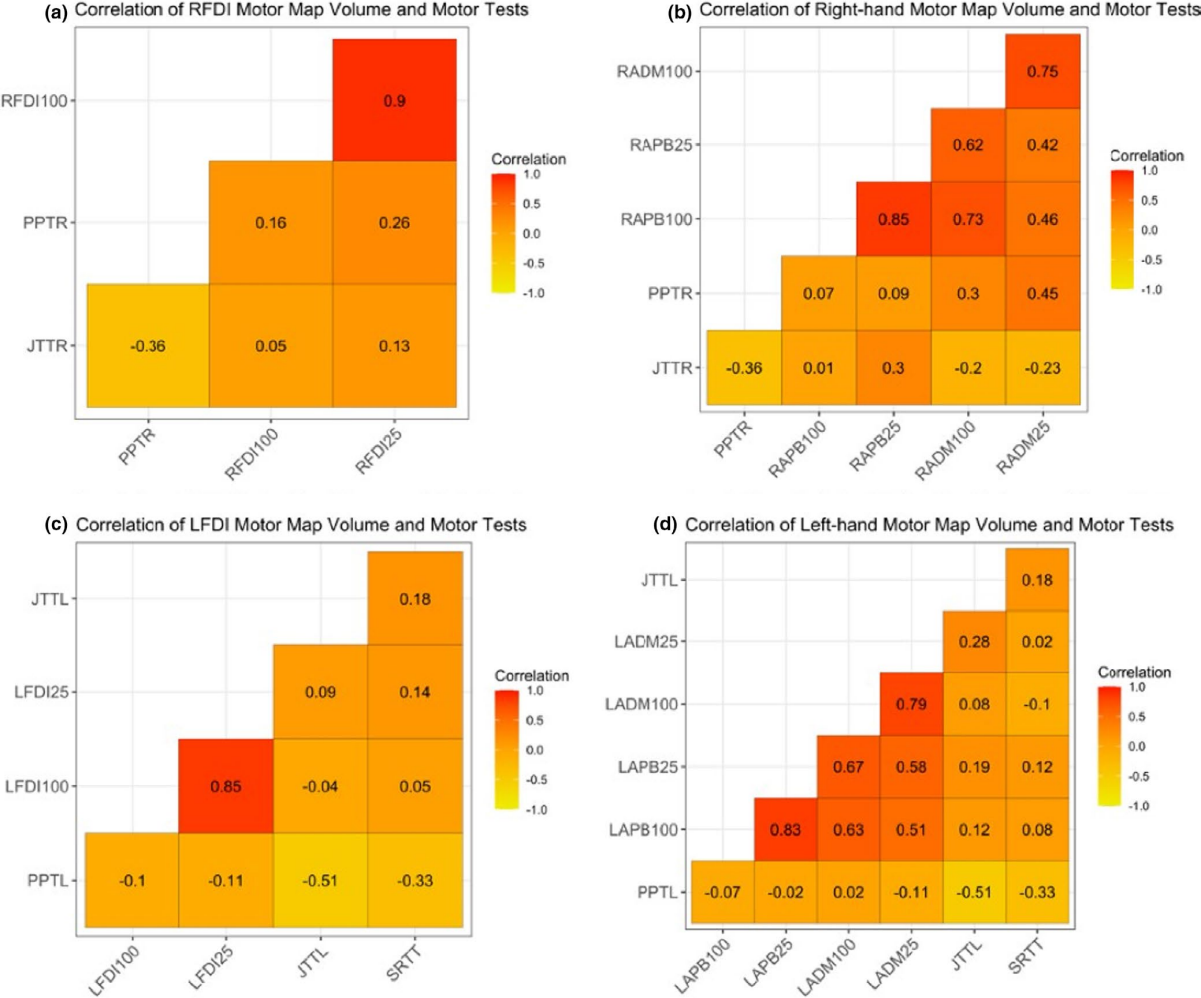
Correlations between motor map volume at subset percentiles with motor tasks in the right‐ and left‐hand muscles. Correlations of subset percentiles of the right‐hand tested muscle (RFDI) map volume and right‐hand motor scores (PPT_R_ and JTT_R_) (a) and of secondary right‐hand muscles map volume and right‐hand motor scores (PPT_R_ and JTT_R_) (b). Correlations of subset percentiles of the left‐hand tested muscle (LFDI) map volume and left‐hand motor scores (PPT_L_, JTT_L_, and SRTT) (c) and of secondary left‐hand muscles map volume and left‐hand motor scores (PPT_L_, JTT_L_, and SRTT) (d). PPT_R_, Purdue Pegboard Task scores in the right‐hand; PPT_L_, Purdue Pegboard Task scores in the left‐hand; JTT_R_, Jebsen Taylor Task scores in the right‐hand; JTT_L_, Jebsen Taylor Task scores in the left‐hand; SRTT, Serial Reaction Time Task; FDI, First dorsal interosseous (tested muscle), secondary muscles; APB, abductor pollicis brevis; ADM, abductor digiti minimi. Arbitrary subset excitability regions chosen (100%, 75%, 50%, 25%). Heat map: Correlations 1.0 (red) to −1.0 (yellow). No significant correlations were found between motor map volume at subset excitability regions with motor tasks in either hand

Correlations between motor map volume and PPT are summarized in Figure 5. Map volume of the right‐hand muscles did not correlate with PPT_R_ scores at any subset region (Figure 5a and b) and volume of the left‐hand muscles did not correlate with PPT_L_ scores at any excitability region (Figure 5c and d).

### 3.8 Secondary motor performance

Neither JTT or SRTT were consistently correlated with map area or volume in either primary hand muscles (FDI) or secondary hand muscles (APB and ADM) across excitability regions in the right (Figure 3) or left (Figure 5) hand.

### 3.9 Euclidian distance of COG

**FIGURE 6:**
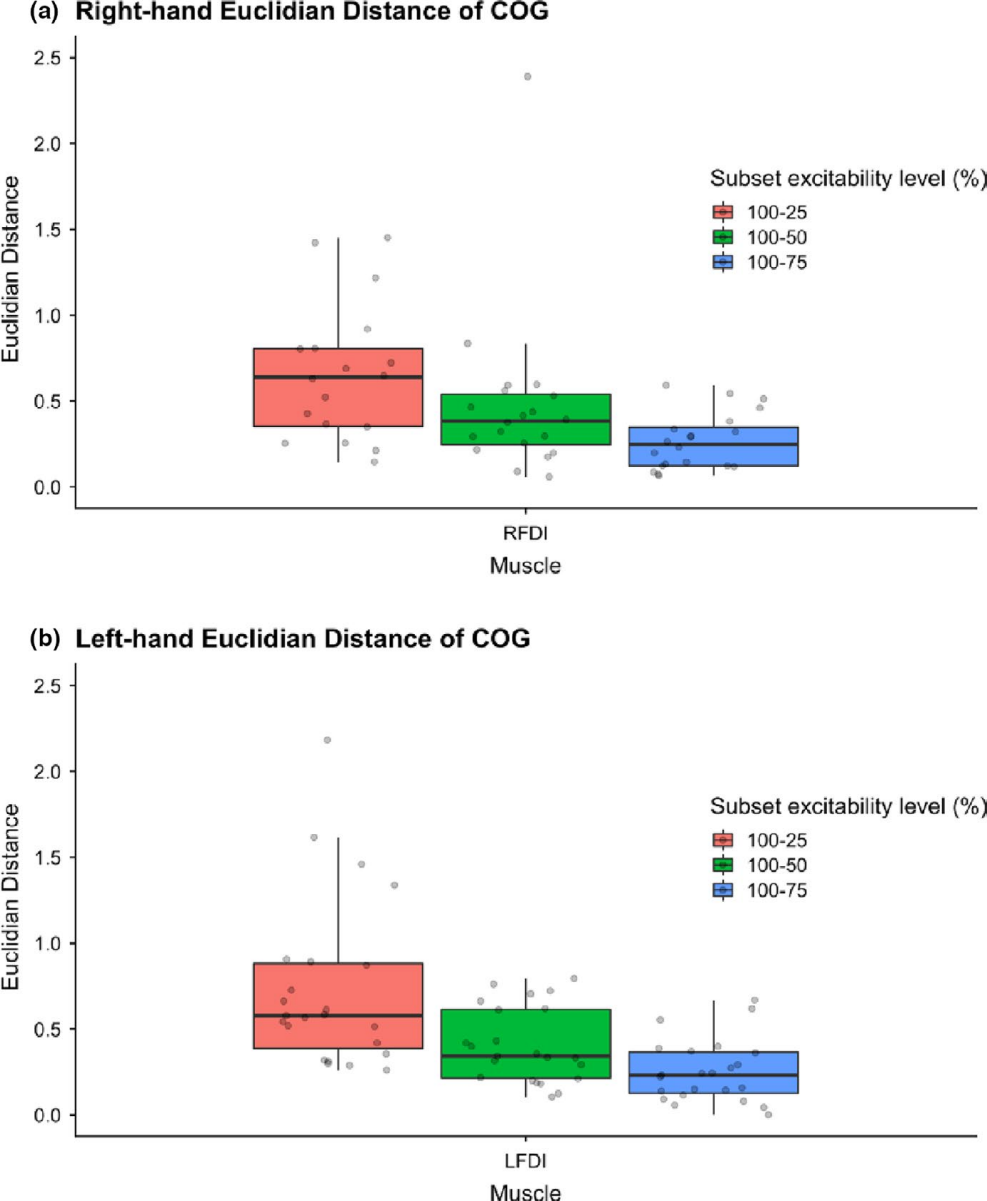
Euclidian distance of COG between subset excitability regions of FDI motor maps. Euclidian distance of COG between subset excitability regions (100–75%, 100–50%, 100–25%) in the right‐hand (a) and left‐hand (b) in the *x*–*y* plane (*z* = 0). Pink = Euclidian distance of COG between excitability regions 100 and 25%; Green = Euclidian distance of COG between subsets 100 and 50%; Blue = Euclidian distance of COG between subsets 100 and 75%. COG, Center of gravity; FDI, First dorsal interosseous. Euclidian distances were significantly different across subset excitability regions (*p* < 0.001)

**FIGURE 7:**
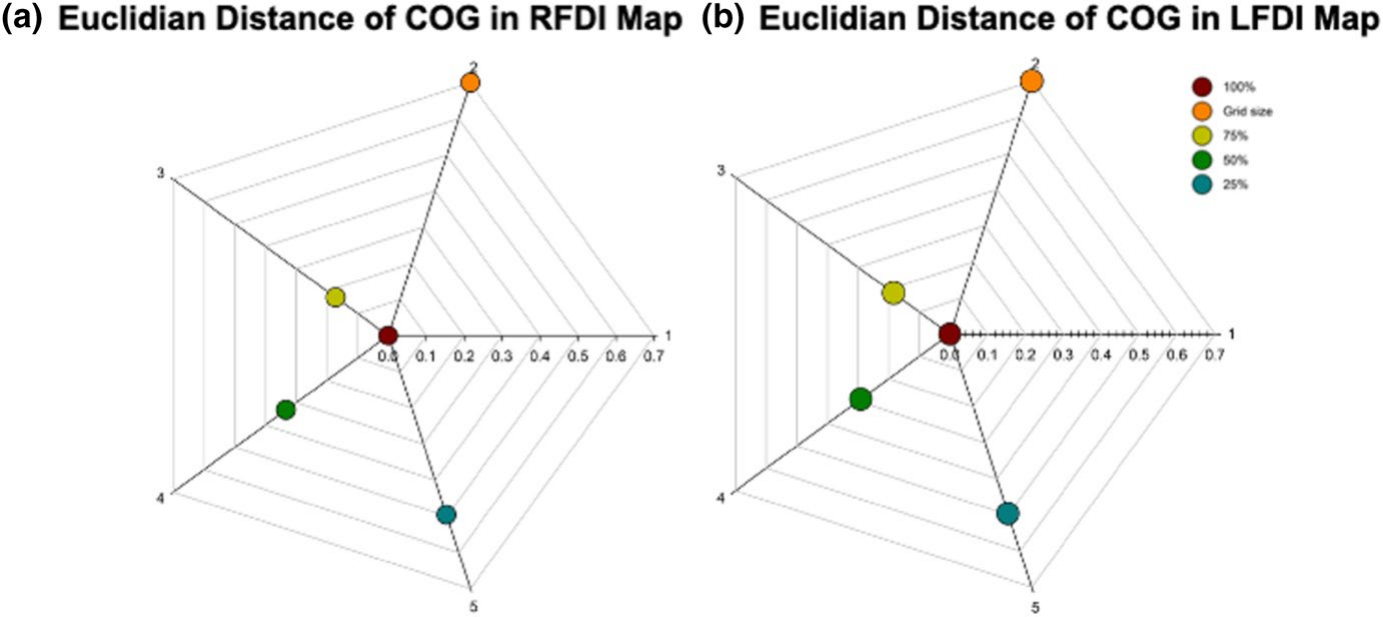
Euclidian distance of COG in the FDI muscle at subset excitability regions. Euclidian distance of COG in the right‐hand FDI motor map (a) and left‐hand FDI motor map (b) across subset excitability regions (100%, 75%, 50%, 25%) in the *x–y* plane (*z* = 0). Dark red = COG at 100%; Light green = Euclidian distance of COG between subsets 100 and 75%; Turquoise = Euclidian distance of COG between subsets 100 and 50%. Blue = Euclidian distance of COG between excitability regions 100 and 25%. Orange = Grid size. Axis = Grid‐point spacing (7 mm). COG, Center of gravity; FDI, First dorsal interosseous

Descriptive statistics of Euclidian distance of COG are reported in Table 2. The Euclidian distance increased from the largest excitability region (R100%) to the smallest excitability region (R25%) (Figure 6). There was no difference between the Euclidian distance of COG at subset excitability regions between the right‐ and left‐hemispheres (R100‐R75% *Z* = −0.299, *p* = 0.765; R100‐R50% *Z* = −0.040, *p* = 0.968; R100‐R25% *Z* = −0.308, *p* = 0.758). Both hemispheres showed differences in Euclidian distances between subset excitability regions (Figure 7). Differences were seen between the right‐hand (R100‐R75%, R100‐R50%, R100‐R25%) (*F* = 25.934, *p* < 0.001) and left‐hand mean Euclidian distance of COG (Kendall's *W* = 0.629, χ^2^ = 28.932, *p* < 0.001). Mean Euclidian distance of COG shifted within the TMS grid‐resolution (7 mm) at subset excitability regions as depicted in Figure 7.

## 4 DISCUSSION

We used robotic TMS to generate detailed bilateral motor maps of multiple hand muscles in typically developing adolescents and compared the characteristics of full and subset excitability regions of motor maps across hemispheres, muscles, and individual factors including motor performance. We observed hemisphere‐specific relationships between subset map areas and motor function. Robotic motor mapping is a potentially valuable tool in the study of motor system neurophysiology, development, and plasticity but multiple factors must be considered in study design and interpretation.

Muscle representations in M1 are well organized and representative of individual muscle control. TMS motor mapping is a safe, well‐tolerated method to explore these representations in vivo. However, previous studies have shown variable results (Brasil‐Neto et al., 1992; Mortifee et al., 1994; Thickbroom et al., 1998; Wilson et al., 1993), the ideal map characteristics and outcomes to quantify neurophysiology and plasticity are yet to be determined, and studies in the developing brain are lacking. Using predetermined subset excitability regions or “slices” of motor maps appears to reveal distinct motor map information while reducing variability. These smaller subset excitability regions may alleviate larger variability in MEP amplitudes observed at the perimeter of the map. Uy et al. (2002) were amongst the first to investigate TMS motor maps using subset excitability regions. Contrary to their findings, we found significant differences in map area and volume across subset excitability regions. This may relate to differences in our methodology such as grid‐size, age of participants, and mapping approach. That these smaller subset maps correlated with motor performance in the dominant hand in a dose‐dependent fashion suggests behavioral significance and may relate well‐studied lateralized differences in the excitability of motor system projections and handedness (Triggs et al., 1994). This observation may also relate to the fact children demonstrate a high degree of laterality in hand performance, both behaviorally and in regards to MEP thresholds of the dominant and non‐dominant motor cortex (Cicinelli et al., 1997). While the smaller number of responsive points at smaller excitability regions and arbitrary nature of the cut‐offs chosen should also be considered, subset motor mapping may be a useful tool in exploring developmental and interventional plasticity in children.

We interpret our observation of dose‐dependent subset map correlations with hand function as an indication of physiological relevance. Multiple studies have quantified cortical representations of hand muscles with or without motor learning, though few have explored associations with motor function. Pascual‐Leone et al. (1995) explored M1 excitability and cortical representations of hand muscles in Braille readers where increased cortical representations of FDI were associated with a decrease in cortical representation of ADM, suggesting the cortical representation of the reading finger enlarged at the expense of other fingers. Other studies using simpler, potentially noisier motor maps, have found no associations between map area or volume and non‐dominant hand motor function (Cicinelli et al., 1997; Pascual‐Leone, Nguyet, et al., 1995; Triggs et al., 1994). Our observation that motor function in the right‐hand correlated with the smallest subset excitability (R25%) of FDI map area may suggest stronger correlations between the larger amplitude MEP found within the “peak of the mountain” (Figure 1c and d). Of course, most motor tasks require the coactivation of numerous muscle groups as compared to a single muscle mapped with TMS. However, differences in excitability regions have been previously observed for different motor tasks (Massé‐Alarie et al., 2017) and it may be possible that the fine‐dexterity motor control required in the PPT task is associated with excitability regions of the FDI map area. The PPT task requires precise movement of the FDI muscle and synergy between the FDI and APB muscles to grasp a small peg. Motor tasks such as the JTT, involved both hand and forearm muscles, and may be correlated with other muscles not measured in this study and their associated peaks of map area.

The shifts we observed in Euclidian distances of the COG across subset excitability regions in both the right and left hemispheres were anticipated. Such shifts in the weighted average of the motor map may add additional understanding of motor map physiology or plasticity. COG is associated with large excitability of corticomotor neurons and has been suggested to be helpful in identifying shifts in cortical representations following interventions (Thickbroom et al., 1998; Wilson et al., 1993). While the effects of higher stimulation intensities on MEP amplitudes and muscle activation are well established (Day et al., 1989; Kiers et al., 1993; Rothwell et al., 1991), these are complimented more recently be examinations of COG (van de Ruit & Grey, 2016). Fewer studies have investigated COG within subset excitability regions of motor maps (Massé‐Alarie et al., 2017; Uy et al., 2002). Uy et al. (2002) investigated shifts in COG of three hand muscles (FDI, APB, ADM) across a 2‐week testing period and found that COG shifted an average of 4 mm, slightly less than previously reported (Miranda et al., 1997). Massé‐Alarie et al. (2017) investigated discrete peaks of cortical M1 representations of synergist and antagonist forearm muscles with motor map characteristics measured at rest and during active muscle contractions. No differences were reported between map volume, COG, and number of peaks between muscles while at rest or during activation. Although there are additional reports of COG stability during active TMS motor mapping (Ngomo et al., 2012; van de Ruit & Grey, 2016), COG within discrete peaks has not been reported. Stable COG coordinates (no shift), as previously seen when exploring stimulation intensities on muscle activation and map area (van de Ruit & Grey, 2016), may suggest cortical neurons are equally excitable along the perimeter of a muscle's cortical representation. Given that the perimeter of motor maps may possess more variable responses (Brasil‐Neto et al., 1992), and here where we showed that COG shifts at subset excitability regions, the significance of COG shifting at subset excitability regions may be insightful when quantifying cortical motor map representations.

The use of TMS motor maps before and after interventional modulations of motor function is an appealing application in translational research and clinical trials. Examples may include motor learning studies in healthy and developmental populations as well as therapeutic interventions in persons with neuromotor disorders such as cerebral palsy. Neuromodulatory effects on motor maps may provide insight regarding mechanisms of M1‐plasticity and considering specific excitability regions as described here may increase the utility of this approach. Our findings here of reduced variability and higher correlations with motor function suggest subset maps may be particularly valuable in such interventional studies which are increasingly rapidly in pediatric populations. Noninvasive brain stimulation techniques such as transcranial direct current stimulation (tDCS) (Ciechanski & Kirton, 2017; Cole et al., 2018) have been shown to enhance motor learning in children but underlying mechanisms are not understood. Both traditional 1 × 1 tDCS and much more focused high‐definition tDCS (HD‐tDCS) appear to demonstrate similar effects on motor learning, suggesting tools capable of interrogating finer components of cortical motor representations may be able to determine elements of map plasticity. With such neuromodulatory and other therapeutic approaches now advancing into phase 3 clinical trials in children with cerebral palsy (Hilderley et al., 2019), tools to explore interventional motor plasticity in vivo are desirable.

One limitation of our study is that mapping was based only on the threshold of a single muscle. Individual thresholding of other muscles that would overcome these limitations would have to be balanced with additional time requirements, particularly in children. In part related to this limitation, we also did not investigate additional motor map metrics such as hotspot density and map overlap characteristics between muscles where future studies may be fruitful. Our study was limited to the resting state whereas active motor maps, which have yet to be performed using robotic TMS in children, may be informative of motor neurophysiology. Activation of a muscle to ~10% of its maximum contraction has been shown to reduce current spread, increase cortical map representations, and allow for lower mapping intensities, potentially benefiting TMS studies conducted in children where rest motor thresholds are higher (Levy et al., 1991). Lastly, test–retest reliability of TMS motor mapping is limited, especially in pediatric populations. Variability of TMS motor maps has been investigated by other groups (Cacchio et al., 2009; Carroll et al., 2001; Corneal et al., 2005; Kimiskidis et al., 2004; Malcolm et al., 2006; Mortifee et al., 1994; Wilson et al., 1993). Our group recently evaluated the short‐ and long‐term reliability of robotic TMS motor mapping, including M1 excitability regions, in young healthy adults (Giuffre et al., 2020). Defining session‐to‐session reliability and minimally detectable change will facilitate the utility of TMS motor mapping in clinical populations.

In conclusion, robotic TMS can safely and efficiently quantify neurophysiological characteristics of motor maps in typically developing school‐age adolescents. Specific map characteristics of individual hand muscles may be associated with related fine motor skills. Subset excitability regions of motor maps may reduce variability and improve the ability to detect correlations with behavior.

## CONFLICTS OF INTEREST

The authors declare no conflict of interest related to the content of this paper.

## AUTHOR CONTRIBUTIONS

Giuffre A: Conceptualization, Methodology, Formal Analysis, Ethics, Recruiting, Investigation, Data collection, Supervision Writing‐Original Draft. Zewdie E: Conceptualization, Methodology, Formal Analysis, Ethics, Recruiting, Investigation, Data collection, Writing‐Manuscript & Editing. Carlson HL: Conceptualization, Methodology, Formal Analysis, Investigation, Data collection, Writing‐Manuscript & Editing. Wrightson JG: Conceptualization, Methodology, Formal Analysis, Ethics, Investigation, Data collection, Writing‐Manuscript & Editing. Kuo H‐C: Conceptualization, Methodology, Formal Analysis, Ethics, Recruiting, Investigation, Data collection, Writing‐Manuscript & Editing. Cole L: Conceptualization, Methodology, Formal Analysis, Ethics, Recruiting, Investigation, Data collection, Writing‐Manuscript & Editing. Kirton A: Conceptualization, Methodology, Ethics, Investigation, Writing‐Original Draft, Supervision.
